# Leaf chlorophyll parameters and photosynthetic characteristic variations with stand age in a typical desert species (*Haloxylon ammodendron*)

**DOI:** 10.3389/fpls.2022.967849

**Published:** 2022-10-06

**Authors:** Xiao-hui He, Jian-hua Si, Dong-meng Zhou, Chun-lin Wang, Chun-yan Zhao, Bing Jia, Jie Qin, Xing-lin Zhu

**Affiliations:** ^1^ Key Laboratory of Ecohydrology of Inland River Basin, Northwest Institute of Eco-Environment and Resources, Chinese Academy of Sciences, Lanzhou, China; ^2^ Faculty of Resources and Environment, University of Chinese Academy of Sciences, Beijing, China; ^3^ Faculty of Resources and Environment, Baotou Teachers’ College, Inner Mongolia University of Science and Technology, Baotou, China

**Keywords:** *Haloxylon ammodendron*, leaf chlorophyll parameters, maximal net photosynthetic rate (*P*_nmax_), maximum light energy use efficiency (LUE_max_), stand age

## Abstract

As a desert shrub, *Haloxylon ammodendron* combines ecological, economic, and social benefits and plays an important role in the ecological conservation of arid desert areas. Understanding its physiological characteristics and its mechanism of light energy utilization is important for the conservation and utilization of *H. ammodendron*. Therefore, we selected five stands (5-, 11-, 22-, 34-, and 46-year-old) of *H. ammodendron* as research objects in the study and measured their photosynthetic light response curves by a portable open photosynthesis system (Li-6400) with a red-blue light source (6400-02B). Then, we measured the leaf chlorophyll parameters in the laboratory, calculated the photosynthetic characteristics by using Ye Zipiao’s photosynthetic model, analyzed their variation patterns across stand ages, and explored the relationships between leaf chlorophyll parameters and photosynthetic characteristics. The results showed that leaf chlorophyll parameters and photosynthetic characteristics of *H. ammodendron* at different stand ages were significantly different. Chl content, *P*_nmax_, and LUE_max_ of *H. ammodendron* were V-shaped with the increase of stand age. The 5-year-old *H. ammodendron* was in the rapid growth period, synthesized more Chl *a+b* content (8.47 mg g^−1^) only by using a narrower range of light, and the *P_nmax_* and LUE_max_ were the highest with values of 36.21 μmol m^−2^ s^−1^ and 0.0344, respectively. For the 22-year-old *H. ammodendron*, due to environmental stress, the values of Chl *a+b* content, *P*_nmax_, and LUE_max_ were the smallest and were 2.64 mg g^−1^, 25.73 μmol m^−2^ s^−1^, and 0.0264, respectively. For the older *H. ammodendron*, its Chl content, *P*_nmax_, and LUE_max_ were not significantly different and tended to stabilize but were slightly higher than those of the middle-aged *H. ammodendron*. On the other hand, the other photosynthetic parameters did not show significant variation patterns with stand age, such as *R*_d_, AQE, LSP, LCP, and *I*_L-sat_. In addition, we found that the relationships between Chl *a+b* content and *P*_nmax_ and between Chl *a+b* content and LUE_max_ were highly correlated, except for the older *H. ammodendron*. Thus, using leaf chlorophyll content as a proxy for photosynthetic capacity and light use efficiency should be considered with caution. This work will provide a scientific reference for the sustainable management of desert ecosystems and vegetation restoration in sandy areas.

## Introduction

*Haloxylon ammodendron* (C. A. Meyer) Bunge is a multistemmed shrub, which has been widely planted in desert areas for its strong ecological adaptability, windbreak and sand fixation ability, and high economic value. It has played an important role in performing important ecosystem functions, maintaining the structure and function of the desert ecosystem, and maintaining an important ecological security barrier along the northern border of China (Su et al., 2007; Maina and Wang, 2015). However, in recent decades, large-scale decline and death of both nature and cultivated *H. ammodendron* were widespread in arid desert areas in northwest China, such as the Ganjiahu Haloxylon Forest National Nature Reserve, Gurbantunggut Desert, desert-oasis ecotone of Zhangye and Minqin, Alaska Plateau, and so on (Yu and Wang, 2018; Wang et al., 2021), which affected the sustainability of the ecosystem (Li et al., 2007). The physiological and ecological changes and sensitivity to the environment of *H. ammodendron* have become one of the key issues that researchers are paying attention to.

Photosynthesis is not only the core component of the carbon cycle in terrestrial ecosystems and the initial driving force of materials and energy cycles on earth but also has a key role in maintaining the carbon–oxygen balance of the atmosphere (Sellers et al., 1997; Zhang et al., 2020; Qian et al., 2021). Leaf chlorophyll (Chl), being the most important and abundant pigment in the plant photosystem (PS), contributes to the conversion of solar radiation into chemical energy (Croft et al., 2020; Qian et al., 2021). Photosynthetic characteristics can reflect the plant’s photosynthetic capacity, physiological and ecological adaptation patterns to different environments, and light utilization strategies (Li et al., 2010). They also may be a diagnostic index of the forest succession and ecological recovery status (Eschenbach et al., 1998; Yang et al., 2008). For trees, the transition from juvenile to adult and then to old may take many years (Merilo et al., 2009). Throughout the growth process, plants have optimized the use of light energy by regulating chlorophyll contents (Chl *a*, Chl *b*, and Chl *a+b*) and ratio (Chl *a/b*), which allows plants to continuously adapt to their environment and promote plant growth (Li et al., 2018). Chl varies significantly across leaf position, nitrogen supply, growing season, leaf ages, and so on (Yang et al., 2014; Li et al., 2018; Croft et al., 2020; Zhang et al., 2020; Zhang et al., 2021a). Net photosynthesis has a decreased trend with increasing tree age or tree size (Kull and Koppel, 1987; Bond, 2000; Greenwood et al., 2008). Zhang et al. (2021a) found that the younger plants would be more shade-tolerant than the older plants by comparing the photosynthetic response of 1- and 4-year-old *savin* juniper plants (Zhang et al., 2021b). In the Alaska Plateau, *H. ammodendron* has been cultivated continuously for more than 40 years. During growth, we suspect that some physiological changes may occur, such as the ability to synthesize photosynthetic pigments and photosynthetic capacity. Perhaps this change is relatively subtle, but it may lead to significant variation in carbon and water exchange in trees of different stand classes (Goulden et al., 1996). Recently, studies on *H. ammodendron* mainly focused on drought resistance, salt stress tolerance, water utilization, and so on (Xu and Li, 2006; Zhou et al., 2017; Wang et al., 2021; Zhou et al., 2022). Nevertheless, up to now, little is known about how Chl and the photosynthetic characteristic of *H. ammodendron* vary with stand age.

In addition, light use efficiency (LUE), as a key parameter in estimating gross primary production (GPP), has been widely studied by scholars and measured by different methods (Farquhar et al., 1980; Ahl et al., 2004; Wang et al., 2010; Guan et al., 2021; Wellington et al., 2022). On the LUE-based GPP estimation model, LUE_max_ was treated as a constant for each biome type (Muramatsu et al., 2014). However, LUE_max_ varies with plant type, canopy density, age, and nutrient conditions (Turner et al., 2003; Wang et al., 2010; Wellington et al., 2022). Regrettably, the accurate simulation of LUE_max_ is still an issue that needs to be addressed. Scientists hypothesized a relationship between chlorophyll content and GPP and confirmed that chlorophyll content is a better proxy for photosynthetic capacity than leaf N (Croft et al., 2017; Qian et al., 2021). This idea is innovative, so we ponder whether chlorophyll content can be used as a proxy for LUE_max_.

Thus, the objectives of this paper are to address the following: 1) compare the changes of leaf chlorophyll (Chl *a*, Chl *b*, Chl *a+b*, and Chl *a/b*) and photosynthetic characteristics (*P*_nmax_, LUE_max_) for *H. ammodendron* at five different stand ages (5-, 11-, 22-, 34-, and 46-year-old) and 2) explore the relationship between leaf chlorophyll and photosynthetic characteristics and verify whether leaf chlorophyll content can be used as a proxy for LUE_max_. This study helps us to clearly know the photosynthetic change rules of *H. ammodendron* with growing years and understand the physiological characteristics and the mechanism of light energy utilization, which is important for ecological restoration in extremely arid areas as well as for providing data and useful information for global carbon calculation.

## Materials and methods

### Site description

The study area is located in Yabulai Town (101°52'E–103°33'E, 39°08'N–40°18'N, mean elevation of 1,585 m), Alxa Right Banner, Alxa League, in Inner Mongolia Autonomous Region of China. It has a typical dry continental climate, the average annual precipitation is 84 mm, and the average annual evaporation is 3,225.7 mm. The region has a large temperature difference between winter and summer, and the average temperature is −9.2°C in January and 25.8°C in July. It has ample sunshine, and the average annual sunshine is 3,000–3,400 h. Due to a worse natural environment, the vegetation cover is low, and the vegetation is mainly composed of super-xerophytic and xerophytic shrubs and subshrubs and xerophytic perennial and annual herbs. After more than 40 years of artificial cultivation, *H. ammodendron* has become the most important, widely planted and distributed sand-fixing species and plays an important role in restoring the local ecological environment.

### Field sampling

**Figure 1:**
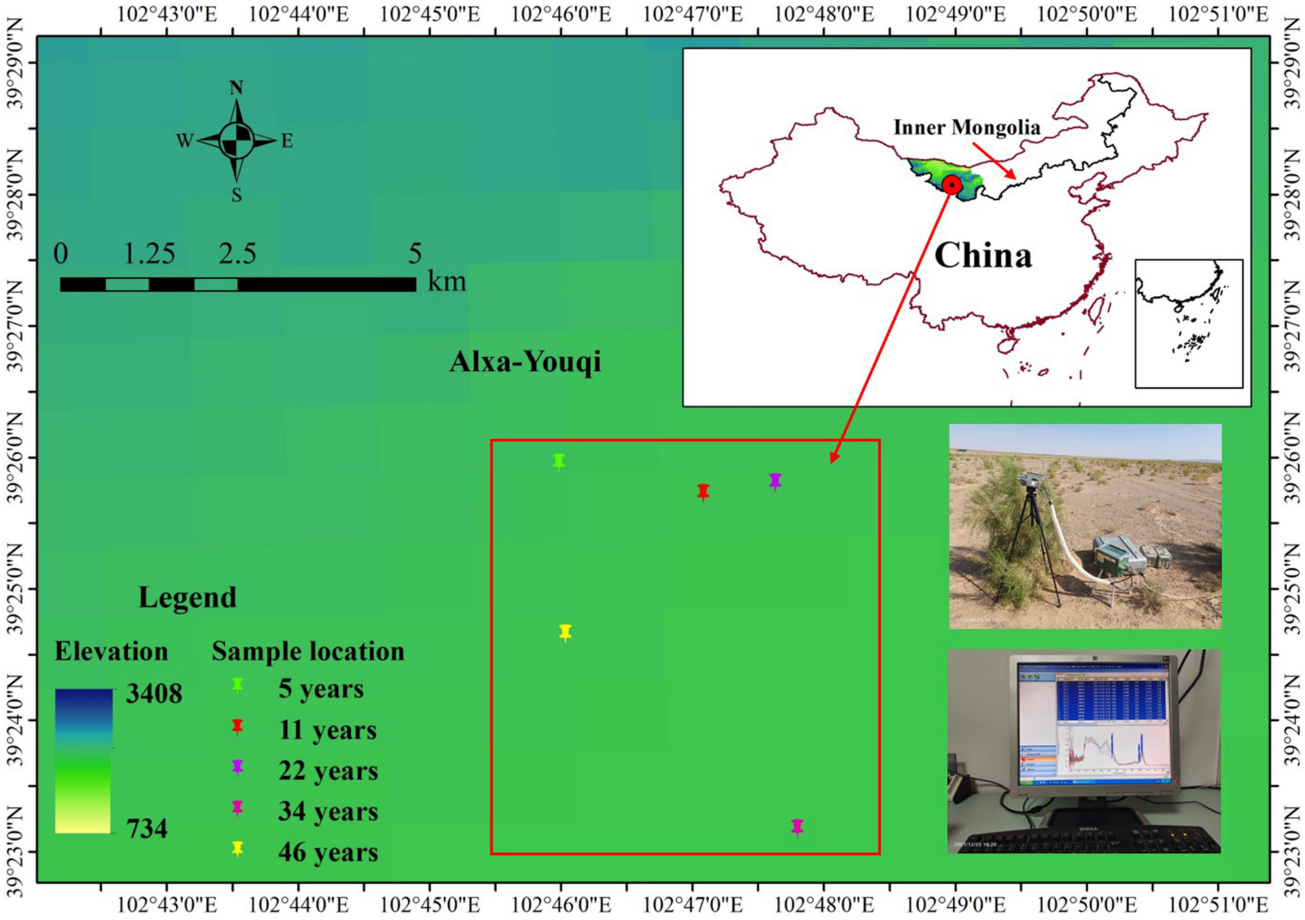
Site locations in Alxa League, China. These were 5, 11, 22, 34, and 46 years of the *Haloxylon ammodendron* samples, respectively.

The field study was performed on the basis of the actual survey and investigation and used the method of substitution of space for time. The stand sequence of *H. ammodendron* was represented by five even-aged stands of different ages: 5, 11, 22, 34, and 46 years old (age indicates how many years a tree has grown since it was planted). These stands are located in the southeastern Badain Jaran Desert, and they are very close to each other (**Figure 1**). They have the same planting density (230 trees per hectare, the most widely used planting density in the study area). They grew on similar soil (sandy soil with loose soil structure) and experience a similar climate. The groundwater level is about 100 m. In addition, *H. ammodendron* was given manual watering for the first 3 years after being planted, after which there was no other human interference. A 50-cm × 50-cm sample plot was established in each stand. No other shrubs grew in the sample plot and annual herbs were distributed sporadically. Three healthy, well-grown *H. ammodendron* trees with little variation were selected from the sample plot in each stand separately. We investigated their location (longitude, latitude, and altitude), crown breadth, diameter, and plant height (**Table 1**). The assimilating branches with better growth and full sunlight were selected from the upper part of each selected *H. ammodendron*, which were tagged as samples for the photosynthetic light response curves and leaf chlorophyll content measurements.

**Table 1:** The growth index of *Haloxylon ammodendron* with different stand ages.

Stand age	Longitude	Latitude	Altitude/m	Crown breadth/cm	Diameter/cm	Plant height/cm
5	102°45′59”	39°25′57”	1,211	62.67 ± 7.88^a^ × 71.00 ± 4.58^a^	2.05 ± 0.44^a^	70.33 ± 2.33^a^
11	102°47′05”	39°25′43”	1,191	142.50 ± 15.88^b^ × 170.00 ± 11.55^b^	9.85 ± 1.41^b^	166.00 ± 8.72^b^
22	102°47′38”	39°25′48”	1,183	175.00 ± 2.89b^c^ × 211.67 ± 8.33^b^	11.76 ± 0.72^bc^	181.67 ± 6.01^b^
34	102°47′48”	39°23′10”	1,178	208.33 ± 13.64^c^ × 216.67 ± 23.33^b^	13.92 ± 0.41^bc^	265.00 ± 14.36^c^
46	102°46′02”	39°24′39”	1,195	366.67 ± 36.67^d^ × 330.00 ± 35.12^c^	16.83 ± 1.95^c^	396.67 ± 3.33^d^

The statistics are mean value ± SE (standard error); different letters denote significant differences at the 0.05 level.

### Gas exchange measurements

The experiment was conducted in August and September of 2021. Each stand was collected two times, three samples were collected each time, and three replicates were collected for each sample. The photosynthetic light response curves of the leaf photosynthesis of *H. ammodendron* were measured by a portable open photosynthesis system (Li-6400; Li-Cor, Inc., NE, USA) with a red-blue light source (6400-02B). Measurements were carried out between 10:00 and 14:00 h. Firstly, we selected 6 to 10 assimilating branches and spread them in the leaf chamber. After the assimilating branches were clamped, the surrounding of the leaf chamber was sealed with plasticine to avoid air leakage. A tripod was used to fix the analyzer to ensure that the assimilating branches were in their original growth position. Secondly, adequate light induction of 0.5–1 h was performed on each assimilated branch before the measurement. Finally, the CO_2_ concentration was held at 400 μmol mol^−1^ by using a CO_2_ small cylinder, with a stepwise photosynthetic photon flux density of 2,000, 1,800, 1,500, 1,200, 1,000, 800, 600, 400, 200, 150, 120, 100, 75, 50, 25, and 0 μmol m^−2^ s^−1^; leaf temperature of 25°C, flow rate of 500 μmol s^−1^; and relative humidity kept between 30% and 50%. A complete photosynthetic light response curve approximately 1 h was carried out.

### Measurement of photosynthetic area and leaf chlorophyll content

After gas exchange measurements, all assimilating branches enclosed in the leaf chamber were carefully cut off for photosynthetic area measurement and chlorophyll content determination.

The assimilating branches were special and could not fill the whole leaf chamber; thus, the total surface area of the assimilating branches could be regarded as the photosynthetic effective area. The assimilating branch is similar to the cylinder, so the vernier calipers (0.05 mm) were used to determine the diameter, and the total area in the leaf chamber can be calculated according to the formula of calculating the surface area of the cylinder (because the leaf chamber is illuminated on one side, the actual photosynthetic area is one-half of the calculated area) (Chen and Black, 1992). The photosynthetic rate was corrected according to the calculated actual area value.

The leaf area (LA) was calculated by the following formula (1):


(1) $$LA\;=\;\frac{2\pi \sqrt{3}}{3}\;d\cdot n$$

Where *d* is the average diameter of assimilating branches and *n* is the number of assimilating branches within the leaf chamber.

After measuring the area, the samples were placed in the car refrigerator (0°C) before being transported to the laboratory and stored at −70°C. We measured Chl content by the following steps: 1) 120 ml of distilled water and 480 ml of acetone were mixed thoroughly; 2) fresh leaves were cleaned; 0.1 g was weighed, cut up, and then 1 ml of distilled water was added and powered and transferred to a 10-ml glass test tube; 3) the volume was adjusted to 10 ml using the solution mixed in step (1) and the solution was allowed to stand for 3–6 h in a dark environment; and 4) 1 ml of the solution extract was taken to the cuvette, and the absorbance values at 663 and 645 nm were measured by using a spectrophotometer and recorded as *A*_663_ and *A*_645_, respectively.


(2) $$\text{Chl }a\text{ content }\left(\text{mg }{\text{g}}^{-1}\right)\;=\;12.7\times \;A_{663}\;-\;2.69\;\times \;A_{645}$$

(3) $$\text{Chl }b\text{ content }\left(\text{mg }{\text{g}}^{-1}\right)\;=\;22.9\;\times \;A_{645}\;-\;4.68\;\times \;A_{663}$$

(4) $$\text{Chl }a\;+\;b\text{ content }\left(\text{mg }{\text{g}}^{-1}\right)\;=\text{ Chl }a\;+\text{ Chl }b$$

(5) Chl a/b ratio = Chl a content / Chl b content

### Analytical model

A non-asymptotic model was developed and tested by Ye to better characterize the light response of photosynthesis (Ye, 2007; Ye et al., 2013; Ye et al., 2020). The formula was as follows:


(6) $$Pn=\alpha \frac{1-\beta I}{1+\gamma I}I-R_{d}$$

The saturation irradiance (*I*_sat_) corresponding to the maximum net photosynthetic rate (*P*_nmax_) can be calculated by formula (7) and formula (8):


(7) $$I_{sat}=\frac{\sqrt{(\beta +\gamma )/\beta }-I}{\gamma }$$

(8) $$P_{\text{nmax}}=\alpha {\left(\frac{\sqrt{\beta +\gamma }-\sqrt{\beta }}{\gamma }\right)}^{2}-R_{d}$$

*α* is the initial slope of the light response curve of photosynthesis, *β* is the photoinhibition coefficient, *γ* is the saturation coefficient, *I* is the irradiance, *P*_n_ is the photosynthetic rate, and *R*_d_ is the dark respiratory rate.

According to the definition of LUE and formula (1), we can derive formula (9):


(9) $$LUE=\frac{P_{\text{n}}}{I}=\alpha \frac{1-\beta I}{1+\gamma I}-\frac{R_{d}}{I}$$

The saturation irradiance (*I*_L-sat_), corresponding to the leaf maximum light energy use efficiency (LUE_max_), can be calculated as follows:


(10) $$I_{L-sat}=\frac{1}{\sqrt{\frac{\alpha \left(\beta +\gamma )\right)}{R_{d}}}-\gamma }$$

(11) $$LUE_{\text{max}}=\alpha \frac{1-\beta I_{L-sat}}{1+\gamma I_{L-sat}}-\frac{R_{d}}{I_{L-sat}}$$

### Statistical analysis

The light response curves and light energy use efficiency were processed by the photosynthetic model fitting software provided by Ye Zipiao. One-way analysis of variance (ANOVA) with *post-hoc* Duncan’s multiple comparison was used to test the differences in leaf chlorophyll and photosynthetic parameters for different stand ages. We used Spearman’s correlation analysis to analyze the relationship between leaf chlorophyll and photosynthesis. Then, we used linear regression analyses to explore the relationship changes with stand age. All analyses were conducted by the software SPSS25 (SPSS Inc., Chicago, IL, USA). All figures were produced in OriginPro 2021 (OriginLab Corp., Northampton, MA, USA). The significance level was set at *P<*0.05.

## Results

### Distribution and variations in leaf chlorophyll content and ratio

**Table 2** illustrates the distribution of leaf chlorophyll parameters, namely, Chl *a*, Chl *b*, Chl *a*+*b*, and Chl *a*/*b.* In the samples, Chl *a* distribution was in the range of 2.24–7.02 mg g^−1^, with a mean value of 4.78 mg g^−1^; Chl *b* was 0.40–2.19 mg g^−1^, with a mean value of 1.21 mg g^−1^; Chl *a*+*b* was 2.64–8.47 mg g^−1^, with a mean value of 5.99 mg g^−1^; and Chl *a/b* was more than 2.25-fold and less than 5.65-fold, with an average value about 4.15. Regardless of leaf chlorophyll content and ratio, the coefficients of variation (CVs) were all approximately 0.25 (**Table 2**).

**Table 2:** Statistics of leaf content (mg g^−1^) and ratio for *Haloxylon ammodendron*.

Leaf chlorophyll	Min	Max	Mean	SD	SE	CV
Chl *a*	2.24	7.02	4.78	1.24	0.23	0.26
Chl *b*	0.40	2.19	1.21	0.37	0.07	0.30
Chl *a*+*b*	2.64	8.47	5.99	1.47	0.27	0.25
Chl *a*/*b*	2.25	5.65	4.15	0.83	0.15	0.20

Min, minimum; Max, maximum; SD, standard deviation; SE, standard error; CV, coefficient of variation.

There were significant differences in Chl *a*, Chl *b*, Chl *a*+*b*, and Chl *a*/*b* among *H. ammodendron* at different stand ages (*P*< 0.05): for Chl *a* content, 5-year-old > 11-year-old > 34-year-old > 46-year-old > 22-year-old (**Figure 4A**); for Chl *b* content, 34-year-old > 5-year-old > 46-year-old > 11-year-old > 22-year-old (**Figure 4B**); and for Chl *a+b* content, 5-year-old > 11-year-old > 34-year-old > 46-year-old > 11-year-old (**Figure 4C**), exactly the same trend as the Chl *a* content. With increasing planting years, leaf chlorophyll content first decreased, then increased, and finally stabilized, and the values of the 22-year-old stand were the lowest. The Chl *a* and Chl *a+b* contents were the highest in the 5-year-old stand, while Chl *b* content was the highest in the 34-year-old stand. However, Chl *a/b* did not follow the pattern of leaf chlorophyll content changes. Chl *a*/*b* was the lowest in the 34-year-old stand, and there were no significant differences at 5-, 11-, and 22-year-old stands (*P*< 0.05). This was evidenced where Chl *a*/*b* was higher in the younger stand compared to the older stand (**Figure 4D**).

### Variations in photosynthetic characteristics

**Figure 2:**
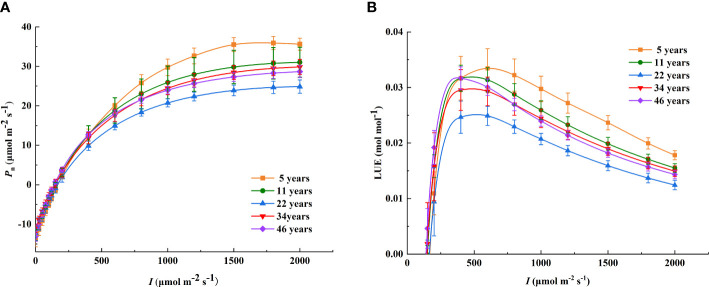
The light response curves of *P*_n_ and LUE at different stand ages. **(A)** The light response curves of *P*_n_; **(B)** the light response curves of LUE. The different colored lines show *Haloxylon ammodendron* with different stand ages. *I*, photosynthetic photon flux density; *P*_n_, net photosynthetic rate; LUE, light use efficiency.

The model selected in this study well simulated the photosynthetic light response curves of *H. ammodendron*, the determining coefficients all reaching a significant level, with *R* ^2^ >0.99 (*P*< 0.001) (**Table 3**). When *I* <250 μmol m^−2^ s^−1^, *P*_n_ was basically the same at different stand ages, and with the enhancement of *I*, significant differences in *P*_n_ appeared among the different stand ages (**Figure 2**). *P*_nmax_ was the highest in the 5-year-old *H. ammodendron*, decreased in the 11-year-old *H. ammodendron*, and reached a valley in the 22-year-old *H. ammodendron*, and then increased in the 34-year-old *H. ammodendron*; the differences between the light response curves of the 34- and 46-year-old *H. ammodendron* were small (**Figure 2** and **Table 3**). There were no significant differences in *P*_nmax_ between the 5- and 11-year-old stands and between the 34- and 46-year-old stands (*P*< 0.05). Above all, the photosynthetic capacity of *H. ammodendron* was related to the stand age: the younger *H. ammodendron* > the older *H. ammodendron* > the middle-aged *H. ammodendron*.

**Table 3:** Statistics of the photosynthetic characteristics of *Haloxylon ammodendron* at different stand ages.

Stand age	*P*_nmax_ (μmol m^−2^ s^−1^)	LSP (μmol m^−2^ s^−1^)	LCP (μmol m^−2^ s^−1^)	*R*_d_ (μmol m^−2^ s^−1^)	AQY (μmol CO_2_ μmol^−1^)	*R* ^2^
5	36.21 ± 1.79^a^	1,873.73 ± 89.65^a^	172.31 ± 8.88^a^	13.60 ± 2.17^a^	0.0805 ± 0.0126^a^	0.9982
11	32.38 ± 2.03^a^	–^ab^	151.25 ± 3.73^a^	12.72 ± 0.59^a^	0.0851 ± 0.0044^a^	0.9969
22	25.73 ± 1.99^b^	–^ab^	167.39 ± 22.27^a^	12.30 ± 1.82^a^	0.0761 ± 0.0105^a^	0.9938
34	30.18 ± 2.05^ab^	–^ab^	152.00 ± 15.38^a^	11.12 ± 0.94^a^	0.0759 ± 0.0072^a^	0.9941
46	30.46 ± 2.34^ab^	–^ab^	140.95 ± 8.11^a^	13.05 ± 8.11^a^	0.0923 ± 0.0080^a^	0.9955

The statistics are mean value ± SE (standard error); –: the light saturation point exceeded 2,000 μmol m^−2^ s^−1^, and no light saturation point, because 2,000 μmol m^−2^ s^−1^ is the maximum light intensity under natural conditions in mid-latitudes. The same letters, not significant; different letters, significant at the 0.05 level.

P_nmax_, maximum photosynthesis; LSP, light saturation point; LCP, light compensation point; AQY, apparent quantum yield; R_d_, dark respiratory rate; R^2^, determining coefficient.

Furthermore, the light saturation point (LSP) of the 5-year-old *H. ammodendron* was 1,873.73 μmol m^−2^ s^−1^, and the others exceeded 2,000 μmol m^−2^ s^−1^. There were no significant differences in light compensation point (LCP) among the different stand ages of *H. ammodendron*; however, the LCP showed a tendency to decrease with the increase of stand age. Similarly, both *R*_d_ and apparent quantum yield (AQY) did not have significant differences among all stand ages of *H. ammodendron* (*P*< 0.05). *R*_d_ tended to decrease with the increase of stand age, while AQY did not show a significant change pattern with the increase of stand age. The minimum AQY was the 34-year-old *H. ammodendron* with a value of 0.0759, and the maximum AQY was the 46-year-old *H. ammodendron* with a value of 0.0923.

### Variations in LUE

As shown in **Figure 2B**, under the condition of low light intensity, LUE increased rapidly and peaked and then decreased as *I* increased. Moreover, the light response curves of light use efficiency were significantly different at different stand ages of *H. ammodendron*. The light intensity required to reach the maximum LUE was different for different stand ages. Compared to the other age classes of *H. ammodendron*, the LUE_max_ of *H. ammodendron* at age 5 was the highest, and the corresponding *I*_L-sat_ was also the highest, with values of 0.0344 mol mol^−1^ and 604.01 μmol m^−2^ s^−1^, respectively. The *I*_L-sat_ corresponding to the LUE_max_ of the 46-year-old stand was minimal, and the values of *I*_L-sat_ and LUE_max_ were 427.62 μmol m^−2^ s^−1^ and 0.0320 mol mol^−1^, respectively. Notably, the LUE_max_ of the 22-year-old *H. ammodendron* was 0.0264, which was the smallest among all stand ages. As the stand age increased, LUE_max_ showed the same trend of decreasing and then increasing similar to *P*_nmax_. However, there was no significant difference (*P*< 0.05) at different stand ages. The *I*_L-sat_ of *H. ammodendron* corresponding to LUE_max_ ranged from 427.62 to 604.01 μmol m^−2^ s^−1^ at each age and tended to decrease with the increase of stand age (**Table 4**).

**Table 4:** The maximum light use efficiency and saturation irradiance for *Haloxylon ammodendron* at different stand ages.

Stand age	LUE_max_ (mol mol^−1^)	*I*_L-sat_ (μmol m^−2^ s^−1^)
5	0.0344 ± 0.0036^a^	604.01 ± 49.21^a^
11	0.0321 ± 0.0023^a^	476.05 ± 11.26^ab^
22	0.0264 ± 0.0022^a^	506.56 ± 65.79^ab^
34	0.0310 ± 0.0036^a^	489.84 ± 50.78^ab^
46	0.0320 ± 0.0019^a^	427.62 ± 25.02^b^

LUE_max_, leaf maximum light energy use efficiency; I_L-sat_, saturation irradiance.

### The relationships between leaf chlorophyll parameters and photosynthetic characteristics

**Figure 3:**
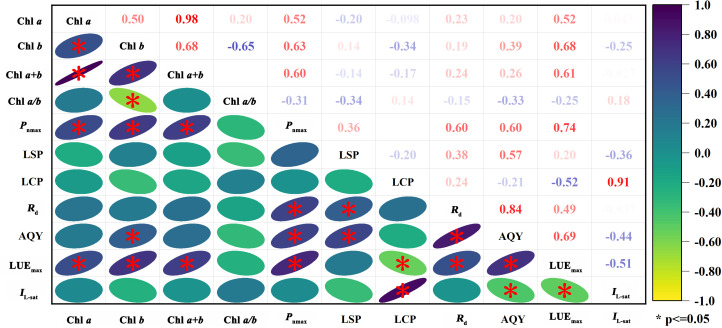
Heatmap of Spearman correlation coefficients between leaf chlorophyll parameters and photosynthetic characteristics (*P* ≤ 0.05).

As shown in **Figure 3**, we found positive relationships between Chl *a* and *P*_nmax_ and between Chl *a* and LUE_max_, and the values of the correlation coefficients were 0.52 and 0.52, respectively. Chl *a* was weakly correlated with *R*_d_ and AQY, did not correlate with *I*_L-sat_, and had a weak negative correlation with LSP and LCP (*P* ≤ 0.05). Chl *b* was significantly positively correlated with *P*_nmax_, LUE_max_, and AQY, and the *R* ^2^ values were 0.63, 0.68, and 0.39, respectively. Chl *b* was weakly correlated with LSP, *R*_d_, and AQY and weakly negatively correlated with LCP and *I*_L-sat_. Chl *a*+*b* was significantly positively correlated with *P*_nmax_ and LUE_max_, both with *R* ^2^ values of 0.60 and 0.61, respectively. Chl *a*+*b* had weak positive correlations with *R*_d_ and AQY, weak negative correlations with LSP and LCP, and no correlation with *I*_L-sat_. Chl *a/b* had an insignificant correlation with photosynthetic characteristics, weak positive correlations with LCP and *I*_L-sat_, and negative correlations with *P*_nmax_, LUE_max_, LSP, *R*_d_, and AQY. The results indicated that leaf chlorophyll contents (Chl *a*, Chl *b*, and Chl *a+b*) had significant effects on *P*_nmax_ and LUE_max_ of *H. ammodendron*.

In addition, as shown in **Figure 6**, there were significant positive correlations between Chl *a+b* content and *P*_nmax_ and between Chl *a+b* content and LUE_max_ at the stage of younger and middle age. The correlation was weak in the older age stage, and this was even a negative correlation. Therefore, we believed that the stand age of *H. ammodendron* may have influenced their relationships between Chl *a+b* content and *P*_nmax_ and between Chl *a+b* content and LUE_max_.

## Discussion

### Impacts of stand age variations in leaf chlorophyll parameters

**Figure 4:**
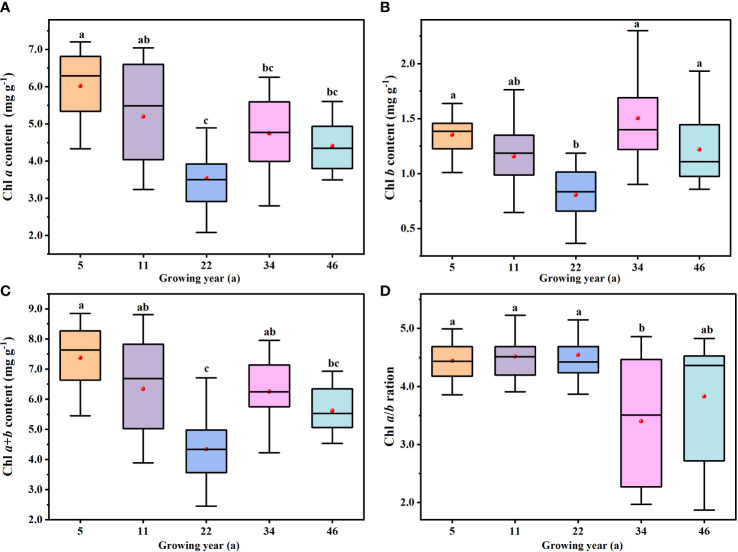
The variations in leaf chlorophyll content and ratio at different stand ages. **(A–D)** Calculated in Chl *a*, Chl *b*, Chl *a*+*b*, and Chl *a*/*b*, respectively. The black lines across the boxes are median values, and the red points represent the mean values. The same letters denote no significant difference among the different stand ages of *Haloxylon ammodendron* (*P*< 0.05).

Leaf chlorophyll content and ratio were analyzed to explore differences among stand ages in light acclimation (Zhang et al., 2021b) and to reflect the use efficiency of direct radiation and adaptability to the environment (Valladares and Niinemets, 2008; Zhang et al., 2020). In our study, the variation of leaf chlorophyll contents (Chl *a*, Chl *b*, and Chl *a+b*) showed a “V” shape with the increase of the stand age (**Figure 4**). For the 22-year-old *H. ammodendron*, the leaf chlorophyll content was the lowest and formed an inflection point. This may be due to the fact that the 22-year-old *H. ammodendron* was subjected to water stress during growth, and water deficiency affected chlorophyll synthesis and promoted chlorophyll decomposition. Chlorophyll is the initiator of plant production. The chlorophyll content of the younger *H. ammodendron* was higher, which was the basis for its rapid metabolism and growth. Wang et al. (2020) compared the chlorophyll content of five stand ages for *H. ammodendron* in Minqin, Gansu Province. Although there was no significant difference, the Chl content of the middle-aged *H. ammodendron* was the lowest and that of the younger *H. ammodendron* was the highest (Wang et al., 2020). In addition, the Chl content of *H. ammodendron* in Minqin was significantly lower than that in our study. On the contrary, Wang et al. (2021) found that Chl content increased significantly with the increase of *H. ammodendron* planted for years in the typical desert–oasis ecotone of the Hexi Corridor (Wang et al., 2021). This may be related to the higher annual rainfall in Hexi Corridor, and *H. ammodendron* was less affected by water stress.

The older *H. ammodendron* (34- and 46-year-old stands) had higher Chl *b* and larger antennae size for better use of diffused light (Grieco et al., 2015). By investigation, we observed that the crown width and plant height of the older *H. ammodendron* were larger (**Table 1**), many assimilating branches occluded each other, and more dead branches appeared. Therefore, we suggested that *H. ammodendron* can improve the utilization rate of low light and achieve light use efficiency by improving the synthesis of Chl *b* and reducing the ratio of Chl *a* to Chl *b* (index of plant tolerance to sun/shade) (Terashima and Evans, 1988; Kitajima and Hogan, 2003). Chl *a*/*b* in the younger *H. ammodendron* was significantly higher than that in the older *H. ammodendron* (**Figure 4D**). On the contrary, Avramov et al. (2017); Zhang et al. 2021a and Zhang et al. 2021b found that the younger plants had higher Chl content and Chl *a*/*b* than the older plants (Avramov et al., 2017; Zhang et al., 2021b). This may be caused by differences in the study species, ages, growing seasons, or leaf positions (Kitajima and Hogan, 2003; Maina and Wang, 2015; Li et al., 2018; Zhang et al., 2020). In addition, Chl *a/b* was more stable than Chl content during the growth of *H. ammodendron*, which shows some phylogenetic signals (Maina and Wang, 2015).

### Impacts of stand age variations in *P*_nmax_ and LUE_max_

Photosynthetic light response curves are an important way to understand plant photochemical efficiency, and the parameters of the curves can reflect the response of plant photosynthetic mechanisms to the environment. *P*_nmax_ is the average photosynthetic rate observed when the incident light is above the LSP, which represents the maximum photon utilization capability and the net primary productivity of the plants and reflects biomass accumulation (Yang et al., 2008). LUE_max_ is an important parameter for estimating GPP or NPP in ecosystems (Yuan et al., 2014). Studying the differences in LUE_max_ can enhance the accuracy of productivity models and provide a basis for carbon cycle studies at regional and global scales. In our present study, we found that photosynthetic responses of *H. ammodendron* differed over the growing stages, with both *P*_nmax_ and LUE_max_ showing a “V”-shaped pattern.

**Figure 5:**
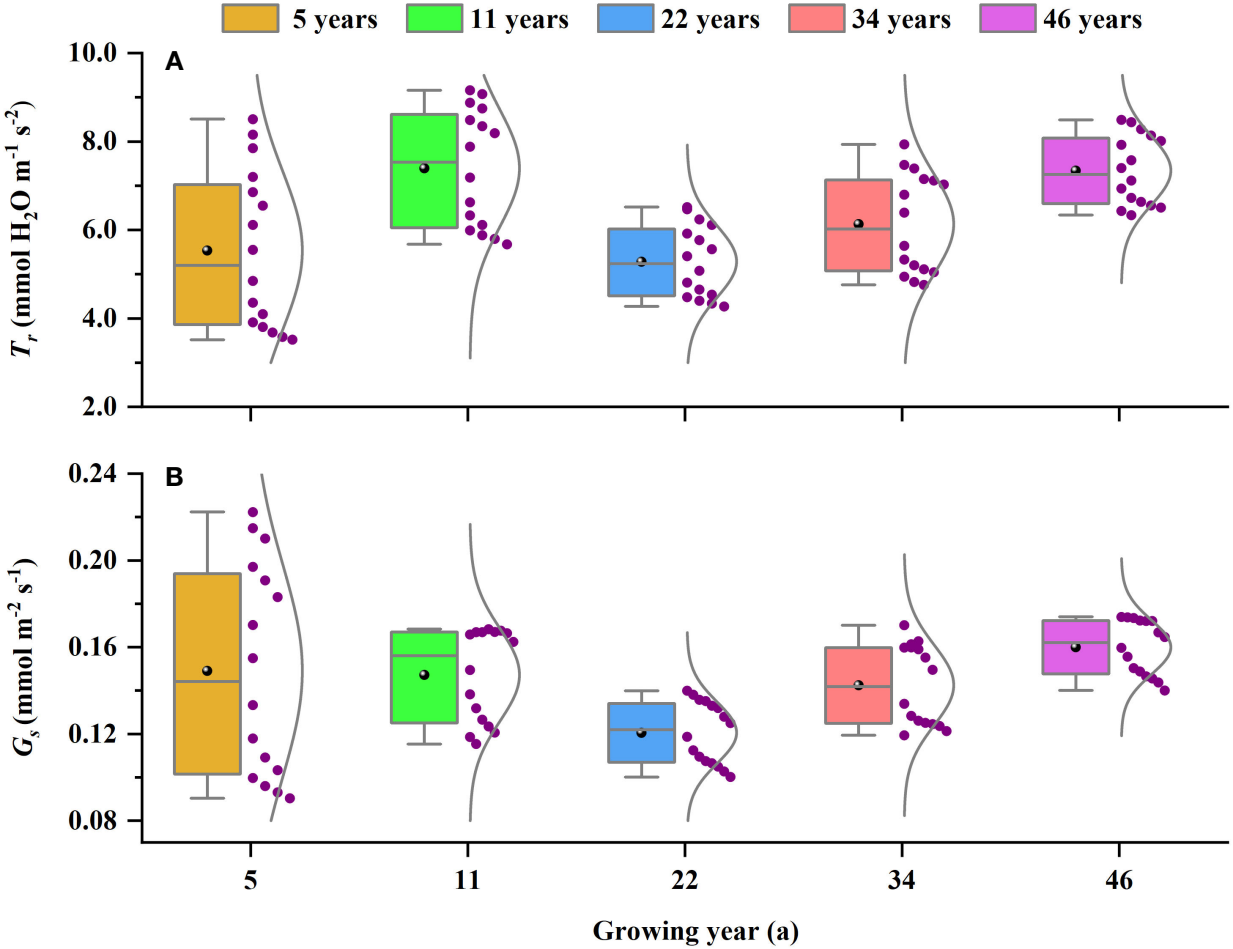
The variations in **(A)** *T*_r_ and **(B)** *G*_s_ at different planting ages. The purple dots represent the values of *T*_r_ and *G*_s_ under different light intensities. *T*_r_, transpiration; *G*_s_, stomatal conductance.

The younger *H. ammodendron* (5- and 11-year-old) were at the peak growth stage, with characteristics such as smaller plants, rapid growth, and well-developed root systems, and the limited precipitation and small soil water content can maintain their growth requirements; thus, they have strong photosynthetic capacity and light use efficiency, which can achieve rapid accumulation of dry matter and thus enhance tolerance to wind and sand flow. At the same time, the *T*_r_ and *G*_s_ of the younger *H. ammodendron* varied widely in response to the changes in light intensity (**Figure 5**), so they have rapid responsiveness to the environmental changes. In addition, the younger *H. ammodendron* only made use of a less narrow range of *I* than the other stand ages, and its LSP was lower and its LCP was higher (**Table 3**), which revealed that using the range of *I* does not effectively determine *P*_nmax_ and LUE_max_. Yang et al. (2008) found that *Ziziphus jujuba* had a higher LUE than *Juglans regia*, but *Z. jujuba* only used the less narrow range of *I* than *J. regia* (Yang et al., 2008).

*Haloxylon ammodendron* may consume much soil water and soil nutrients in the early stage of rapid growth, resulting in a stressed growth environment for the middle-aged *H. ammodendron* (22-year-old). Soil water has an important effect on the activity and quantity of photosynthetic enzymes (Yang et al., 2008); thus, water deficit limited the photosynthetic capacity and LUE (Gao et al., 2016). When subjected to water stress, *H. ammodendron* regulated the physiological characteristics by reducing stomatal conductance, decreasing transpiration (**Figure 5**), and reducing respiratory consumption, to realize the optical protection process (Demmig-Adams and Adams, 2000). In this way, organic matter was accumulated through low photosynthesis for defense and adaptation to adversity. At the same time, *H. ammodendron* was able to expand the range of available light by decreasing LSP and increasing LCP under severe drought conditions (**Table 3**), which improves its ability to use resources efficiently. This is consistent with the conclusion of Yang et al. (2018). The *P*_nmax_ and LUE_max_ increased in the older *H. ammodendron* (34- and 46-year-old), and there was a decreasing trend with growth. In response to this phenomenon, we believed that there are three main reasons for this phenomenon: firstly, the growth speed of the belowground part of *H. ammodendron* was prior than that of the belowground part (Wei et al., 2007), the main depth that *H. ammodendron* used soil water increased as stand age increased (Zhu and Jia, 2011), and water stress was alleviated by the formation of a more powerful root system in the growth of *H. ammodendron* (34- and 46-year-old), in order to adapt to the arid environment; secondly, degradation of the older *H. ammodendron* (34- and 46-year-old) has occurred, and the ratio of the numbers of live to dead branches of individual *H. ammodendron* decreased, providing opportunities for photosynthesis by live branches which have larger stomatal conductance; thus, the live branches of the older *H. ammodendron* had higher *P*_nmax_ and LUE_max_; thirdly, *H. ammodendron* has developed its own extreme drought tolerance mechanism and photosynthetic regulation strategy in long-term adversity (Gao et al., 2016), and the older *H. ammodendron* plants are able to use a greater range of light and maintain a higher photosynthetic capacity and light energy use efficiency. However, with the increase of stand age, plants have a tendency to senesce, physiological activity decreases, and photosynthetic capacity gradually decreases (Schoettle, 1994).

### Leaf chlorophyll content should be cautiously used as a proxy for LUE_max_

**Figure 6:**
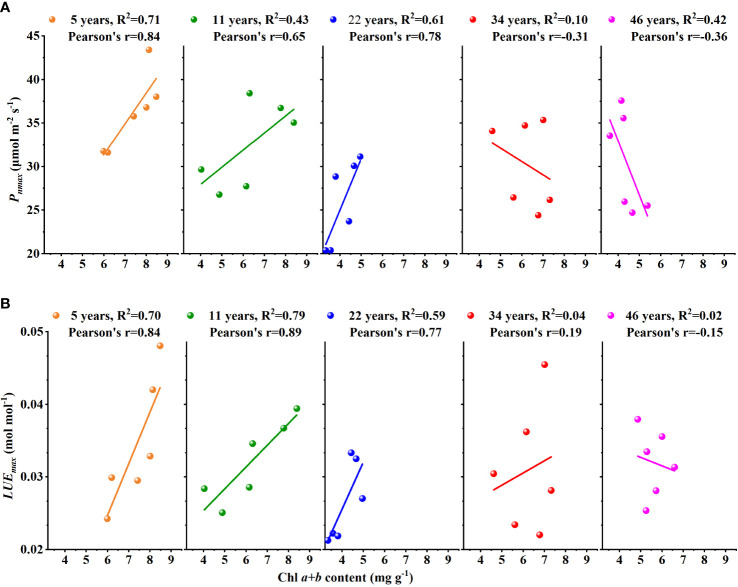
The relationships **(A)** between Chl *a+b* content and *P*_nmax_ and **(B)** between Chl *a+b* content and LUE_max_ changes with the stand age of *Haloxylon ammodendron*.

Chl is a key factor affecting photosynthetic capacity and vegetation productivity and can be accurately retrieved, so it can be used as a proxy for *V*_cmax25_ at large scales to resolve the uncertainty in *V*_cmax25_ in C cycle modeling (Croft et al., 2013; Croft et al., 2017; Qian et al., 2021). LUE_max_ is also an important parameter for productivity estimation. The accurate simulation of LUE_max_ is the basis for quantifying spatial and temporal changes in productivity and the global carbon cycle. Thus, we hope to establish the relationship between LUE_max_ and Chl content. So, we tested the correlation coefficient between Chl *a+b* content and LUE_max_ in different stand ages of *H. ammodendron.* Contrary to expectations, the *R* ^2^ tended to decrease with the increase of stand ages (**Figure 6**) and *R* ^2^<0.2 in the older *H. ammodendron* (34- and 46-year-old). We believed that Chl might be used as a proxy for LUE_max_ in the younger *H. ammodendron* (5- and 11-year-old) and middle-aged *H. ammodendron* (22-year-old), but the older *H. ammodendron* cannot. In the past, scholars found a similar phenomenon when studying the relationship between PRI and LUE. This relationship was affected by leaf age, and the reason was that carotenoid and chlorophyll content had a relatively significant negative correlation on PRI (Penuelas et al., 1995; Gamon et al., 2001; Guo and Trotter, 2004).

## Conclusions

After long-term cultivation, *H. ammodendron* has become a major windbreak and sand-fixing species in the Alaska Plateau. Understanding the physiological features of *H. ammodendron* at different stand ages is helpful for protecting and utilizing *H. ammodendron*, which is important to curb the ecological degradation in arid desert areas. In this study, we reported leaf chlorophyll pigments and photosynthesis variation with stand age. We found that the Chl content, *P*_nmax_, and LUE_max_ of *H. ammodendron* were V-shaped with the increase of stand age. The younger *H. ammodendron* was in the growing stage and had a higher ability of chlorophyll synthesis and photosynthesis; the middle-aged *H. ammodendron* was affected by environmental stress, and it reduced the synthesis of Chl content and maintained life requirements by reducing photosynthesis, which needs proper human intervention. While the older *H. ammodendron* can form a stable adaptation mechanism in long-term growth, its Chl content, *P*_nmax_, and LUE_max_ were not significantly different and tended to stabilize but were slightly higher than those of the middle-aged *H. ammodendron*. In addition, the relationship between Chl *a+b* content and LUE_max_ decreased with the increase of the stand age. Thus, using leaf chlorophyll content as a proxy for photosynthetic capacity and light use efficiency should be considered with caution.

## Data availability statement

The original contributions presented in the study are included in the article/Supplementary Material. Further inquiries can be directed to the corresponding author.

## Author contributions

X-HH conceived the ideas, designed the methodology, participated in the field works, and wrote the manuscript. J-HS designed the methodology and organized the manuscript. D-MZ, C-LW, and JQ participated in the field works and indoor experiments. BJ and C-YZ analyzed the data and results. D-MZ, C-LW, JQ, and X-LZ produced the figures and tables. All authors contributed critically to the article and approved the submitted version.

## Funding

This study was supported by the Major Science and Technology Project in Inner Mongolia Autonomous region of China (No. Zdzx2018057); the Innovation Cross Team Project of Chinese Academy of Sciences, CAS (No. JCTD-2019-19); Transformation Projects of Scientific and Technological Achievements in Inner Mongolia Autonomous region of China (No. 2021CG0046); Science and Technology Research Project of Colleges and Universities in Inner Mongolia Autonomous Region (NJZY21034); and the National Natural Science Foundation of China (No. 42001038).

## Acknowledgments

The authors thank all the members of the Key Laboratory of Eco-Hydrology of Inland River Basin, Northwest Institute of Eco-Environment and Resources, Chinese Academy of Sciences and the Faculty of Resources and Environment, Baotou Teachers’ College, Inner Mongolia University of Science and Technology.

## Conflict of interest

The authors declare that the research was conducted in the absence of any commercial or financial relationships that could be construed as a potential conflict of interest.

The reviewer J-LL declared a shared affiliation, with the authors X-HH, J-HS, D-MZ, C-LW, C-YZ, BJ, JQ, X-LZ to the handling editor at the time of the review.

## Publisher’s note

All claims expressed in this article are solely those of the authors and do not necessarily represent those of their affiliated organizations, or those of the publisher, the editors and the reviewers. Any product that may be evaluated in this article, or claim that may be made by its manufacturer, is not guaranteed or endorsed by the publisher.
